# The Design and Performance Evaluation of an Eye-Tracking System Based on an Electrostatic MEMS Scanning Mirror

**DOI:** 10.3390/mi16060640

**Published:** 2025-05-28

**Authors:** Minqiang Li, Lin Qin, Xiasheng Wang, Jiaojiao Wen, Tong Wu, Xiaoming Huang, Hongbo Yin, Yi Tian, Zhuqing Wang

**Affiliations:** 1 School of Electronic Engineering, Chengdu Technological University, Chengdu 610031, China; minqiangli@163.com; 2 School of Mechanical Engineering, Sichuan University, Chengdu 610065, China; qinlin19583625790@163.com (L.Q.); 2022141410152@stu.scu.edu.cn (X.W.); wenlay5410@foxmail.com (J.W.); wzhuqing@scu.edu.cn (Z.W.); 3 Sichuan Eye Hospital, Aier Eye Hospital Group, Chengdu 610041, China; victortongwu@126.com (T.W.); hxm@tmu.edu.cn (X.H.); 4 Department of Wound Repair, The Second Hospital of Hunan University of Chinese Medicine, Changsha 410011, China

**Keywords:** eye-tracking, scanning mirror, electrostatic actuation, Lissajous scanning

## Abstract

In this paper, we proposed an eye-tracking system featuring a small size and high scanning frequency, utilizing an electrostatic biaxial scanning mirror fabricated through a micro-electro-mechanical system (MEMS) process. A laser beam is directed onto the mirror, and the two axes of the mirror generate a Lissajous scanning pattern within an artificial eyeball. The scanning pattern reflected from the eyeball is detected by a linear photodiode sensor array (LPSA). The direction and rotation angle of the artificial eyeball result in varying grayscale values across a series of pixels detected by the LPSA, in which the average grayscale values change accordingly. By performing a linear fit between different rotation angles of the same eye movement direction and the corresponding grayscale values, we can determine the correlation between the direction of eye movement and the signal magnitude received by the LPSA, thereby enabling precise eye tracking. The results demonstrated that the minimum resolution was 0.6°. This preliminary result indicates that the system has good accuracy. In the future, this eye-tracking system can be integrated into various wearable glasses devices and applied in various fields, including medicine and psychology.

## 1. Introduction

Eye-tracking technology, which observes the eye gaze point [1], has a long and evolving history of research and methodology development dating back to the early 20th century. Initially, direct observation [2] and mechanical recording methods [3] were employed to study eye movements. Over time, various techniques emerged, including the corneal reflection method, electrooculography (EOG) [4], and scleral search coils [5].

In the mid-20th century, advancements in optoelectronic sensing technologies introduced methods such as limbus tracking [6], retinal image-based eye tracking [7], and dual-Purkinje imaging (DPI) [8], significantly enhancing accuracy and usability. Many of these techniques, along with subsequent camera-based systems, leveraged infrared (IR) illumination and detection techniques for robustly determining pupil position or corneal reflections. This approach was favored because IR light provides high contrast and is imperceptible to the human eye, thus minimizing distraction. By the 21st century, camera-based video recording methods became the mainstream approach for eye tracking. Shih et al. [9] proposed a novel method for real-time three-dimensional gaze tracking, employing 3D computer vision technology. This method utilizes multiple cameras and point sources to estimate the optical axis of the user’s eye, yielding 30 gaze measurements per second. They also introduced a straightforward and accurate gaze-tracking calibration method, which required users to briefly fixate (2–3 s) on a target point to estimate the constant angle between the 3D line of sight and the optical axis. Test results demonstrated promising performance for the system, achieving an average estimation error of less than 1 degree. Bobić et al. [10] introduced a camera-based eye-tracking technique by employing image segmentation technology [11]. Pupil coordinates were computed in each successive video frame. Sensitivity (S) and positive predictive values (PP) were used to evaluate the accuracy of camera-based eye tracking. Results revealed that tracking accuracy diminished with subject movement, yielding only S = 87.50% and PP = 88.61%. Tracking errors were attributed to limitations in image-processing capabilities. Nevertheless, camera-based eye movement tracking faces challenges such as high power consumption, low lens resolution, and high development costs [12].

Beyond camera-based eye-tracking technology, scanning-based eye-tracking technology also exists. MEMS mirrors, millimeter-scale vibrating reflective mirrors fabricated through MEMS processes [13,14], offer distinct advantages. Characterized by mirror surfaces precisely controlled within millimeters, MEMS mirrors consume less driving energy, exhibit larger driving displacements, and possess smaller footprints compared to conventionally manufactured mirrors [15]. These features enable the mass production of MEMS mirrors at lower costs. Furthermore, owing to their exceptional performance, MEMS mirrors have found extensive applications across various cutting-edge fields such as lidar, optical communications, and optical coherence tomography imaging [16,17,18,19].

Benefiting from their remarkable capabilities, MEMS mirrors have also found utility in eye tracking [20]. Meyer et al. [21] proposed a novel miniaturized, low-power eye-tracking sensor method. This technique leverages the varying infrared reflectivity across different regions of the eye. A laser beam scattered from the eye surface reflects wavelengths with varying intensities, which are then received and measured by an optical photodiode. Gaze information can then be extracted by interpreting the photodiode signal as an image, utilizing established video-based oculography (VOG) algorithms [22]. This approach offers significant advantages over typical VOG systems, notably high integration capability and low power consumption. Furthermore, it employs a less sophisticated pupil tracking algorithm, thereby reducing computational requirements and power consumption while maintaining a comparable gaze angular resolution compared to traditional VOG sensors. However, the temporal resolution of this eye-tracking sensor is constrained by the frame rate of the retinal projection system, which is currently limited to 60 Hz. Additionally, external light sources may interfere with the captured image, potentially affecting the accuracy of the VOG algorithm.

Addressing the limitations identified in prior studies, this paper proposes an eye-tracking system based on a biaxial MEMS mirror augmented by a novel linear photodiode sensor array (LPSA) as the scanning device. The mirror integrates an electrostatically driven structure fabricated using MEMS techniques. A raster scan model was employed to assess the performance of the MEMS mirror and determine the resonance frequencies of its two axes, thus enabling full-area scanning. The MCU processes the photoelectric signal to determine the eye-tracking system’s parameters. Utilizing a PSD (position-sensitive detector) as a receiver enables high-frequency eye scanning. Tracking resolutions in four directions are calculated via linear fitting of grayscale values. Finally, by comparing predicted grayscale values with actual measurements, the system’s tracking errors are derived. This scanning-based method demonstrates minimal power consumption while providing a broad tracking region.

## 2. Materials and Methods

### 2.1. Electrostatic MEMS Mirror

**Figure 1:**
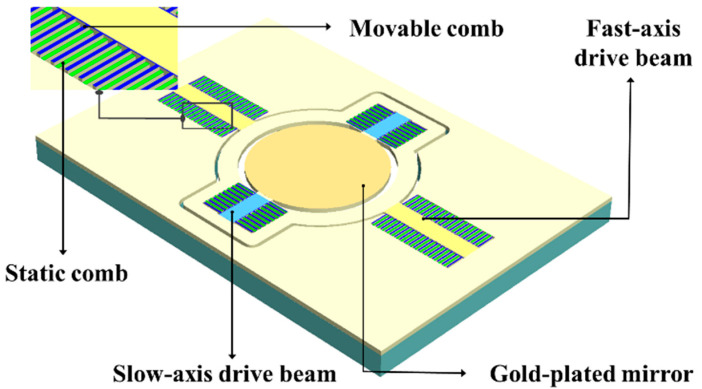
The electrostatic comb structure of the MEMS mirror.

The MEMS mirror, depicted in Figure 1, primarily comprises four components: the fast-axis drive beam, the slow-axis drive beam, the electrostatic combs, and the gold-plated mirror. The static and movable combs are interleaved. The application of a square wave voltage generates an electrostatic force, causing the movable combs to oscillate in simple harmonic motion. To maximize the vibration amplitude, the signal voltage frequency is set to twice the resonant frequency of the driving beam. The voltages applied to the fast and slow axes are independent. The electrostatic comb drive consists of 52 comb pairs. For this experiment, a MEMS galvanometer with a fast-axis resonant frequency of 52.07 kHz and a slow-axis resonant frequency of 4.2 kHz was used. The adopted driving method achieves nanosecond-level response times.

### 2.2. Lissajous Scanning of the MEMS Mirror

**Figure 2:**
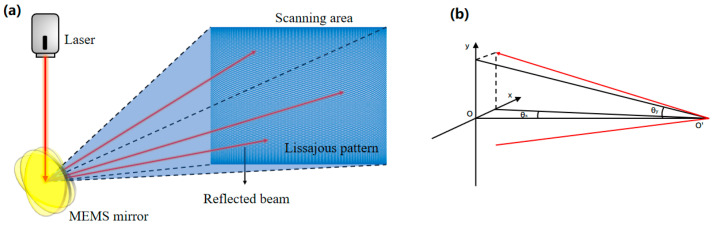
Micromirror scanning mode diagram. (**a**) The Lissajous scanning area based on the MEMS mirror. (**b**) The reflection path of the laser spot.

The MEMS mirror vibrates at its resonant frequency along both its fast and slow axes. The combination of these two simple harmonic vibrations determines the trajectory of the single laser beam reflected from the mirror. Consequently, the scanning pattern is solely dependent on the vibration frequencies of the two axes. This adherence to Lissajous scanning principles means that the observed Lissajous pattern remains constant when the ratio of the fast and slow axis vibration frequencies is constant. The laser-scanned area generated by the MEMS mirror is depicted in Figure 2a.

When the biaxial micromirror operates at resonance, the reflected laser generates sinusoidal scanning along each of the two axial directions:

(1a) $$x=x_{0}\sin(2\pi f_{x}t)\;$$

(1b) $$y=y_{0}\sin(2\pi f_{y}t)$$

where f*_x_* is the frequency of the spot’s simple harmonic vibration parallel to the *x*-axis and f*_y_* is the frequency of its simple harmonic vibration parallel to the *y*-axis. The spot radius is 1.5 mm. Here, *x*_0_ and *y*_0_ denote the *x*- and *y*-axis lengths of the maximum scanning image generated in the scanning plane at a distance of d from the micromirror. The term d represents the total distance from the laser emitter to the MEMS galvanometer mirror and subsequently to the eye surface; it is expressed as follows:

(1c) $$d\cdot \tan\theta_{1}=x_{0}$$

(1d) $$d\cdot \tan\theta_{2}=y_{0}$$

where θ_1_ and θ_2_ are the maximum optical scanning angles of the micromirror in the *x*- and *y*-axes, respectively. The path of the reflected laser light is illustrated in Figure 2b.

### 2.3. The Function Principle of MCU and LPSA

The LPSA used in this experiment is TSL1401CL, a linear photodiode sensor array manufactured by ams AG. This device is housed in an 8-pin package, featuring a central linear array of 128 photodiodes, with an overall length on the centimeter scale. During operation, three pins—serial input (SI), clock signal (CLK), and analog output (AO)—interface with the corresponding pins on the MCU. The SI pin facilitates the transmission of control signals from the MCU to the LPSA. The MCU-generated CLK directly governs the integration time and the duration of a single sampling cycle; higher clock frequencies result in shorter integration and sampling times. The AO pin transmits analog data comprising voltage signals generated from photodetection. These signals are directly connected to the analog-to-digital converter (ADC) input pin.

The main microcontroller unit (MCU) employed in this experiment is STM32F108C8T6. Leveraging the MCU’s general purpose input/output (GPIO) pins, signals are sent to the LPSA to promptly initiate the integration process upon signal reception. Integration time is determined by the clock cycles’ (high and low levels) output by the MCU GPIO at predefined intervals. Once the voltage values from the LPSA sensor have stabilized, the ADC is triggered to capture and convert 128 analog voltage signals into digital signals for storage. Since the resulting digital quantity is 12 bits, which is not optimally suited for single-byte storage, normalization of the converted digital signals is required. In the implemented program, the upper 8 bits of the converted data are extracted as the final result, ensuring precision and streamlining subsequent data output. These data are then transmitted to the host computer via a universal synchronous asynchronous receiver transmitter (USART) for further processing. Due to the LPSA’s total pixel count of 128, optimizing serial transmission rates while preserving signal integrity necessitates transmitting values for every 8 pixels. This process commences from the 4th pixel, collecting values from the 4th, 12th, 20th, …, and 124th pixels as serial input data.

### 2.4. Setup of the Eye-Tracking Test System

**Figure 3:**
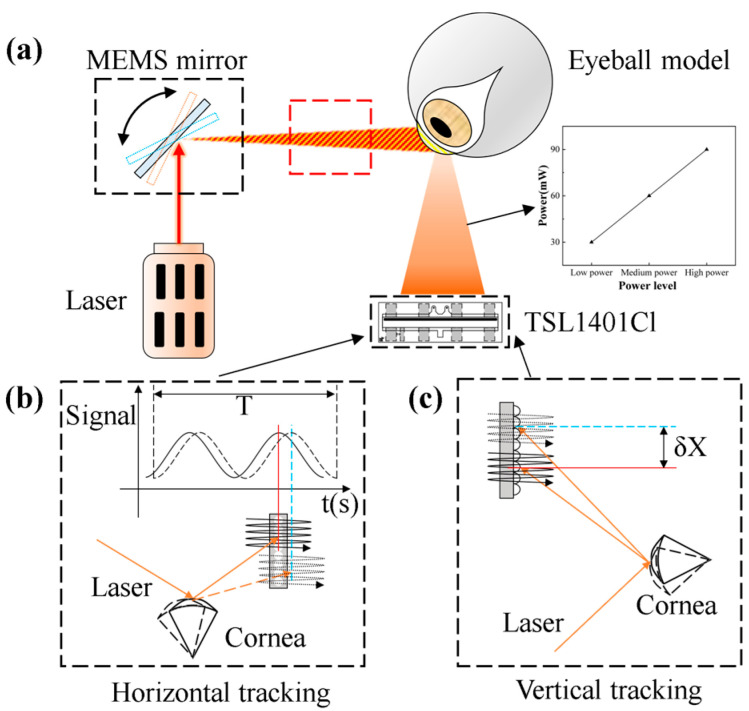
Schematic of the eye-tracking system. (**a**) Model of the eye tracking system (**b**) Horizontal variation (**c**) Vertical variation.

To construct the eye-tracking system, several components were integrated alongside the MEMS mirror. Specifically, a single adjustable power 639 nm laser source (with a spot radius of 1.5 mm), a 25 mm diameter stainless steel eyeball model, and a 2.5 mm diameter black pupil model were employed. The artificial eyeball bearing is symmetrically centered, ensuring that the maximum angle of rotation is consistent in all directions. The laser source’s power was calibrated during the initial setup to meet operational requirements. Data reception and MEMS mirror actuation are managed by two separate MCUs. Figure 3 illustrates the complete system schematic.

**Figure 4:**
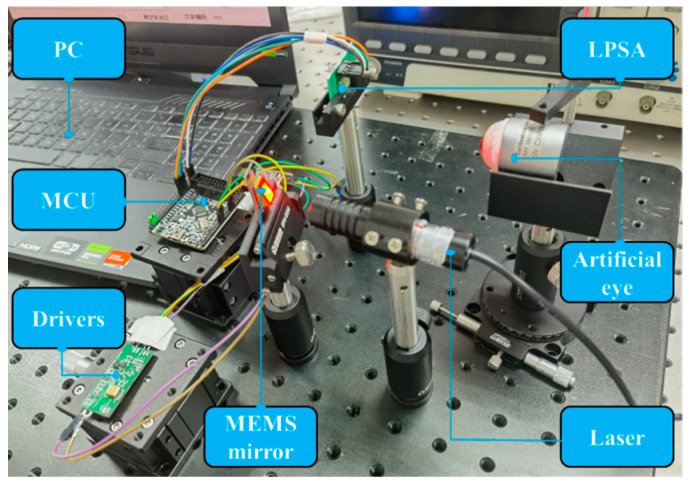
The eye-tracking system.

Signals within the eye-tracking system are categorized as optical and electrical. The optical signals involve near-infrared light emitted by the laser source, which is reflected off the eye model by the MEMS mirror scan and subsequently detected by the LPSA. The electrical signals include control signals essential for system operation, such as the clock signal for LPSA data acquisition and the square wave signal for powering the MEMS mirror. Regarding eye-tracking algorithms, the LPSA supports horizontal and vertical tracking. While Figure 3b,c conceptually illustrate how eye movements cause the reflected raster to shift, the actual discrimination between horizontal, vertical, and oblique movements relies on analyzing characteristic changes in the grayscale intensity profile across the LPSA’s pixels. These intensity profiles, along with changes in the average grayscale value, are detailed for different eye movements in Figure 9 and Section 3.2, forming the basis for identifying the eye’s direction. The overall tracking result is also influenced by system parameters, including distance and laser power. The complete eye-tracking system is depicted in Figure 4.

## 3. Results

### 3.1. Determination of Eye-Tracking System Parameters

**Figure 5:**
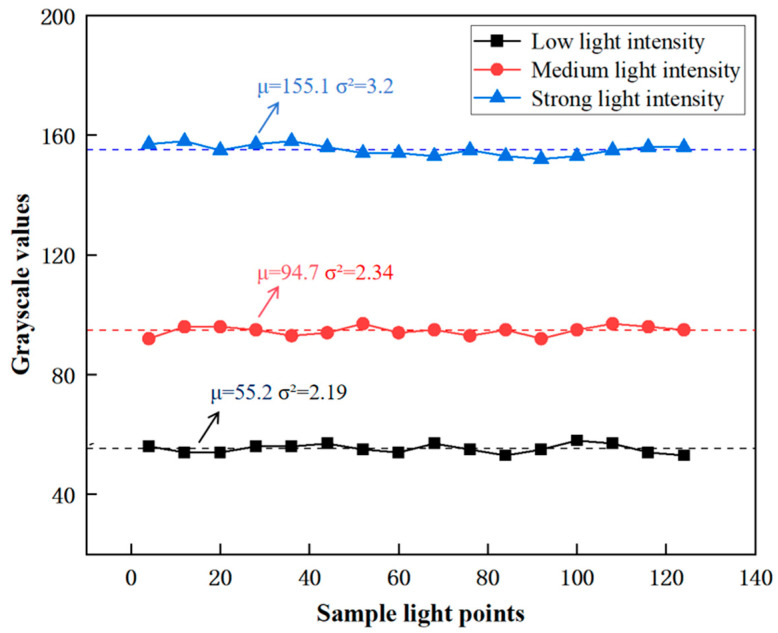
Optoelectronic signals stability test.

During the experimental phase, variations in the signal output from the photoelectric receiver array were initially examined under different laser source powers. This was performed to ascertain system stability and, significantly, explore how changes in light intensity—resulting from these power variations—affect the grayscale values, as illustrated in Figure 5. Our measurements indicated that the LPSA exhibits high stability. Concurrently, we observed that different light intensity settings yielded distinct mean grayscale values: 55.2 in low light, 94.7 in medium light, and 155.1 in bright light [23], demonstrating a clear relationship between light intensity and the recorded grayscale.

Additionally, minimal oscillations were observed in the mean values across the sampled pixels. Variance was calculated to further quantify the measured stability of the LPSA. The variances of the LPSA were 2.19, 2.34, and 3.20 for the three light intensities, respectively. Relative to their corresponding mean grayscale values, the LPSA demonstrated stable performance under diverse lighting conditions. Therefore, the LPSA was deemed suitable for signal reception within the eye-tracking system [24].

**Figure 6:**
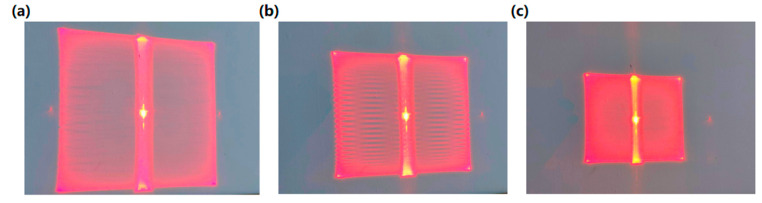
Different scales of scanning results: (**a**) large-scale scanning, (**b**) medium-scale scanning, and (**c**) small-scale scanning.

The dimensions of the MEMS mirror’s scanning region also influence the LPSA’s signal output. By adjusting the applied signal frequency, the scanning range can be modified to be consistent with the resonant driving mechanism of the MEMS mirror. The laser power was adjusted to evaluate the scanning range and optimize signal acquisition, with a 30 mW laser source ultimately selected. The results for large, medium, and small scanning scales are presented in Figure 6.

**Figure 7:**
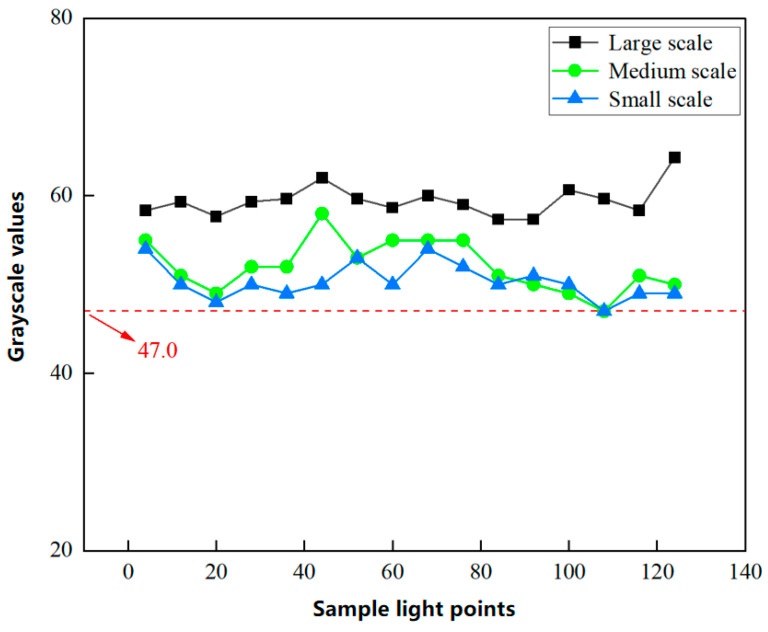
Signal results for large, medium, and small-scale scanning.

Considering the high stability of grayscale values obtained from the LPSA, a single sampling suffices for reliable measurement. Figure 7 displays the signal curves for three scanning scales (large, medium, and small). The large scanning scale yields significantly different results compared to the medium and small scanning scales, particularly concerning average grayscale values. For the medium and small scanning scales, the minimum observed grayscale value was 47, indicating an insufficient dynamic range for grayscale value variation in continuous measurements. This limitation poses a challenge in identifying characteristic curves during eye tracking. In contrast, the large scanning scale exhibits greater fluctuations in grayscale values due to eyeball rotation, thereby providing a wider dynamic range for signal detection. Consequently, the large scanning scale was selected as the optimal scanning region for the eye-tracking system [25].

### 3.2. Signal Change Pattern of Eye-Tracking

In order to verify the feasibility of the study, an eye model based on fisheye orientation is used, and the accuracy of the experiment can be subsequently improved by algorithmic optimization. The maximum rotation angle of the fisheye bearing-based eye model, which was segmented into three angular gradients (10°, 20°, and 30°), is approximately 30°. To ascertain the resolution in each direction, signal variation was independently monitored for top, bottom, left, and right movements.

**Figure 8:**
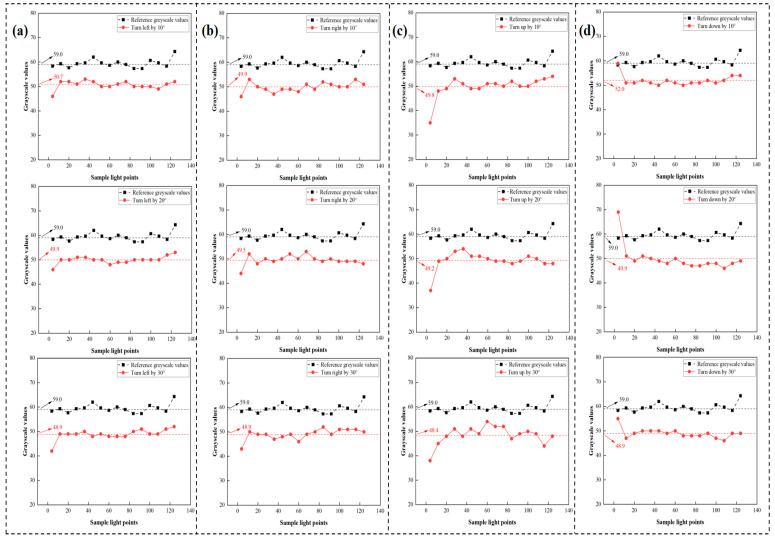
Signal for different angles of the eye model: (**a**) turn left by 10°, 20° and 30°; (**b**) turn right by 10°, 20°, and 30°; (**c**) turn up by 10°, 20°, and 30°; and (**d**) turn down by 10°, 20°, and 30°.

Figure 8a illustrates the LPSA signal output at different angles for leftward movement. A vertical comparison reveals a decrease in signal output amplitude across almost every pixel as the eye model turns left, albeit to varying extents [26]. A horizontal comparison reveals two key findings: First, the average grayscale value decreases with an increasing left-turn angle, as the reflected raster moves farther from the LPSA. Second, regardless of the left-turn angle, the curve’s trajectory remains consistent; that is, the three red lines exhibit essentially the same trend.

The same set of rotation angles was used to evaluate the signal when the eye model turned right, with the results shown in Figure 8b. These results indicate that each sampled pixel exhibits a distinct decrease in grayscale value compared to the left-turn condition. Furthermore, a horizontal comparison for the right turn also shows that the grayscale values of the three curves decrease with increasing rotation angle, and the curves maintain a similar general trend. However, the *rate* of decrease in average grayscale values for right turns is slower compared to left turns, and the overall curve shape exhibits distinct differences.

Consequently, upward and downward rotations were set to the same angular increments as the left and right rotations. Figure 8c shows the LPSA signal output at different angles for upward movement [27]. The signal observed during upward movement is similar to that observed for left and right movements, with grayscale values decreasing by varying amounts across different pixels. However, a horizontal comparison reveals that upward movement differs in having the lowest average grayscale values among the tested directions. Additionally, upward movement produces a distinctive grayscale value curve shape. Lastly, the signal generated by the downward movement of the eye model is depicted in Figure 8d.

The outcomes for downward movement align with the general patterns observed for other rotation directions. First, downward movement results in a decrease in grayscale values, similar to other directions. Second, each rotational angle increment corresponds to a distinct average grayscale value. Third, downward movement causes the LPSA signal to exhibit a unique curve shape. Thus, it is possible to establish the specific direction and angle of rotation based on the grayscale value curve trend and the average grayscale value from the LPSA data [28,29].

### 3.3. Resolution Calculation for Eye-Tracking

Each of the four rotation directions corresponds to a different rate of decrease in the average grayscale value. It is observed from the original LPSA data (exemplified in Figure 8 that the rate at which the average grayscale value changes with rotation angle differs for each of the four cardinal directions. This varying sensitivity is an empirical result of the system’s specific optical configuration. Based on the fitting formula derived from our experimental data, a linear relationship was established between the rotation angle and the change in the average grayscale value. Considering that the LPSA has a resolution of 1 grayscale value, the average grayscale value possesses a minimum resolution of 0.1. Therefore, leveraging this linear relationship, if the average grayscale value changes by one unit due to rotation, the rotation angle can be deduced accordingly. The consistency in the rate of decrease in grayscale values across the four directions further supports this linear model.

**Figure 9:**
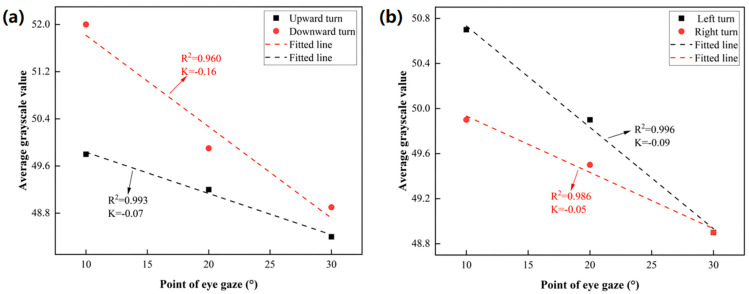
(**a**) Results of the upward- and downward-turn fittings. (**b**) Results of the left- and right-turn fittings.

Here, *x* represents the angle of rotation, while b is the initial gray value without rotation, and k is the rate at which the gray value decreases with the angle. The results of the left- and right-turn fits are shown in Figure 9a.The results of the upward- and downward-turn fits are shown in Figure 9b.

Fitting results for a left turn:

(2a) y=−0.09x+51.63

(2b) $$R^{2}=0.996$$

Fitting results for a right turn:

(2c) y=−0.05x+50.43

(2d) $$R^{2}=0.986$$

Fitting results for an upward turn:

(2e) y=−0.07x+50.53

(2f) $$R^{2}=0.993$$

Fitting results for a downward turn:

(2g) y=−0.16x+53.37

(2h) $$R^{2}=0.960$$

The *R*^2^ of the fitting results exceeds 0.95, indicating a high degree of linearity in the four directions of rotation. The relationship between the inferred resolution p and the slope k is as follows:

(3) $$p=\frac{0.1}{k}$$

The minimum accuracy of the sensor output is 0.1, and the accuracy is sufficient to not be affected by sensor quantization, noise, etc.

### 3.4. Error Calculation for Eye-Tracking

**Figure 10:**
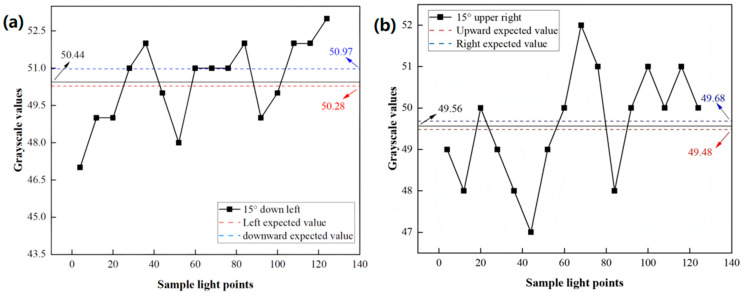
(**a**) Signal curve with a 15° rotation to the down left (**b**) Signal curve with a 15° rotation to the upper right.

As eye-tracking signals are independent across each direction and each exhibits a unique grayscale–angle response profile, tracking accuracy can be simultaneously verified through their combined analysis. For instance, signals from a 15° rotation to the upper right and a 15° rotation to the lower left can be assessed concurrently. This combined verification relies on the approximation that for small angular changes, the horizontal and vertical components of oblique eye rotation contribute quasi-independently to the overall shift in the reflected light pattern across the linear photodiode sensor array (LPSA), thereby influencing the final average grayscale value in a way that can be decomposed using 1D calibration models. Rotation in oblique directions involves coupled horizontal and vertical components; consequently, it is expected to manifest combined characteristics of both. For instance, the average grayscale values should approximate those of the constituent horizontal and vertical movements, and the signal curve should resemble their combined trends. Fitting results indicate an expected mean grayscale value of 49.68 for a 15° rightward rotation and 48.48 for a 15° upward rotation. Figure 10a illustrates the value curve for a 15° upper-right rotation of the eye model [30].

The analysis of these results reveals that the oblique rotation’s observed values deviate from those of its pure components: a decrease of 0.12 relative to the rightward expectation and an increase of 0.08 relative to the upward expectation. These differences manifest within each component’s contribution to the combined signal. Similarly, for a 15° rotation, the expected grayscale values are 50.28 for the leftward direction and 50.97 for the downward direction. The value curve corresponding to a 15° lower-left eye model rotation is depicted in Figure 10b.

Error for a left turn:

(4a) 50.28=−0.09x+51.63

(4b) x=16.78

(4c) ∆=1.78

Error for a right turn:

(4d) 49.68=−0.05x+50.43

(4e) x=17.4

(4f) ∆=2.4

Error for an upward turn:

(4g) 49.48=−0.07x+50.53

(4h) ∆=1.14

Error for a downward turn:

(4i) 50.97=−0.16x+53.37

(4j) ∆=3.31

The mean grayscale level is 50.44, indicating a decrease of 0.53 compared to the downward turn and an increase of 0.16 compared to the left turn.

The error calculation involves substituting the grayscale values into the error calculation formulas for upward, leftward, rightward, and downward rotation, respectively. For instance, the left rotation error is determined by inputting the grayscale value of 50.28 into Equation (4a), yielding a theoretical left rotation angle of 16.78 degrees. The error, calculated as the absolute difference between the theoretical angle of 16.78 degrees and the actual angle of 15 degrees, is found to be 1.78 degrees.

**Figure 11:**
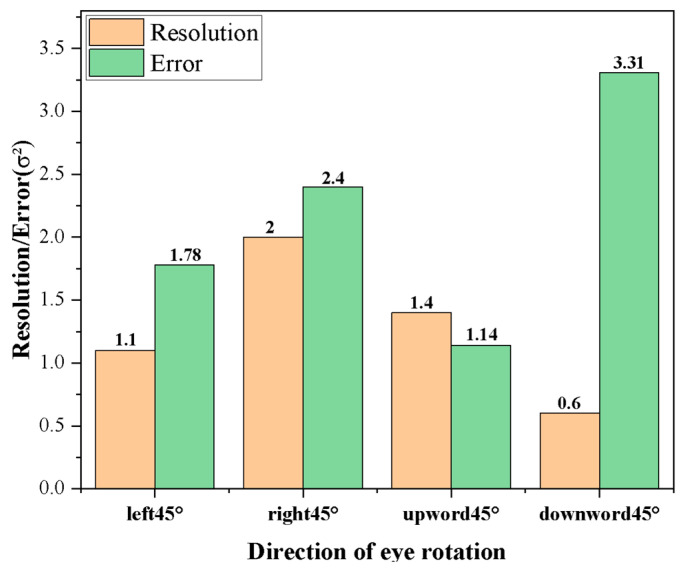
Comparison of error and resolution in each direction.

Finally, the error in the angle of rotation is calculated from the slope of the descent. These results are compared with the resolution in each direction, as depicted in Figure 11. According to the results, the tracking errors are as follows: 1.78° for the left turn, 2.40° for the right turn, 1.14° for the upward turn, and 3.31° for the downward turn. Among them, the downward rotation has the smallest resolution but also the largest tracking error, which is due to the fact that when rotating in a diagonal direction, the single direction will be affected by the rotation factors of the other direction; for example, the downward rotation has the highest gray value, and combined with left rotation, the gray value will be ‘pulled down’ to a certain extent. And because the downward rotation has the largest absolute value of the slope, the tracking error from the calculation is also the largest. In the subsequent experiments, the accuracy can be improved by optimizing the AI algorithm.

## 4. Conclusions

This study developed and evaluated an eye-tracking system utilizing an electrostatic MEMS mirror. Fabricated using standard MEMS techniques, the mirror offers notable advantages in cost-effectiveness and power efficiency. Driven by an electrostatic comb, the mirror generates a Lissajous pattern through its scanning operation. Laser light reflected from the mirror is detected by a position-sensitive detector (LPSA), which is controlled by a microcontroller unit (MCU) for optoelectronic signal conversion. Concurrently, the MCU transmits data to a host computer for subsequent analysis. System stability, quantified by grayscale variance, consistently remained below 3.20. System parameters were optimized based on the LPSA’s signal curve characteristics, determining that a 30 mW laser source and large-area scanning are optimal.

The rotation of the eyeball model in four cardinal directions produced distinct grayscale value curves, each characterized by varying rates of decrease in the average grayscale value. Linear fitting was employed to model the grayscale variation in each direction, demonstrating a strong goodness of fit, with correlation factors (*R*^2^) exceeding 0.95. The tracking resolution, which was inferred from the slope (k), was determined to be 0.6° in the downward direction. To assess tracking accuracy, oblique rotations of 15° in the upper-right and lower-left directions were applied. The results indicated tracking errors of 1.78° for the left turn, 2.40° for the right turn, 1.14° for the upward turn, and 3.31° for the downward turn. Notably, despite the downward-turn resolution being the finest, this direction also exhibited the largest tracking error.

Overall, the findings demonstrate that the electrostatic MEMS mirror reliably generated a stable Lissajous scanning pattern. The eye-tracking system utilizing this mirror achieved high resolution and low tracking error across four directions. This level of accuracy is competitive with, and in some instances surpasses, that of previously reported camera-based systems (e.g., Shih et al. [9], which reported <1° error), particularly when considering the additional benefits of the proposed system. These initial results underscore the significant potential of employing this system in the development of eye-tracking devices. Moreover, the device boasts notable advantages concerning power efficiency, weight, portability, and cost-effectiveness. Consequently, it presents a promising avenue for the future advancement of eye-tracking equipment.

## Data Availability

Data is contained within the article; The original contributions presented in this study are included in the article. Further inquiries can be directed to the corresponding author(s).
